# Multi-Drug-Resistant Elizabethkingia meningoseptica: A Rare Cause of Late-Onset Sepsis in a Preterm Neonate

**DOI:** 10.7759/cureus.34361

**Published:** 2023-01-29

**Authors:** Abdul Wasey Hashmi, Muhammad Ahmad, Muhammad Muneeb Israr, Ibtesam e Fajar, Farid Adnan

**Affiliations:** 1 Internal Medicine, Shalamar Medical and Dental College, Lahore, PAK; 2 Surgery, Bahawal Victoria Hospital, Bahawalpur, PAK; 3 Surgery, Al-Nafees Medical College and Hospital, Islamabad, PAK; 4 Pediatrics, Walsall Manor Hospital, The Royal Wolverhampton NHS Trust, Walsall, GBR

**Keywords:** multi-drug resistance, elizabethkingia, meningitis, preterm neonate, late onset sepsis

## Abstract

Elizabethkingia meningoseptica is a gram-negative bacillus and is a rare cause of opportunistic infections. Literature shows that this gram-negative bacillus may cause early-onset sepsis in neonates and immunocompromised adults; however, it is a rare cause of late-onset sepsis or meningitis in neonates. We hereby delineate a case of a preterm neonate, born at 35 weeks of gestation, presenting to us on the eleventh day after birth, with fever, tachycardia, and delayed reflexes. The neonate was managed in the neonatal intensive care unit (NICU). Initial laboratory tests, blood, and cerebrospinal fluid (CSF) cultures showed evidence of late-onset sepsis due to multi-drug-resistant E. meningoseptica sensitive to vancomycin and ciprofloxacin. The patient completed the antibiotic therapy and was discharged from the hospital. The patient was followed up at one and two months after discharge in the tele-clinic and was thriving well with no complaints.

## Introduction

Elizabethkingia meningoseptica is a gram-negative, nonmotile, non-spore-forming bacillus from the Weeksellaceae family of bacteria and was discovered in 1959 [1]. The bacterium is intrinsically resistant to most β-lactam antibiotics and carbapenems [2]. Elizabethkingia species are widespread and have been isolated from numerous natural sources [1-3]. Moreover, they rarely cause nosocomial infections ranging from neonatal sepsis to complicated urogenital infections in neonates and immunocompromised adults [3]. Literature shows that in adults, it can cause urinary tract infections, meningitis, nosocomial ventilator-associated pneumonia, and bloodstream infections associated with indwelling catheters. However, in neonates, it usually causes early-onset sepsis and meningitis [1-4]. Infections with this bacterium are difficult to manage due to the limited availability of clinical and genetic data, making it liable to misidentification [3]. This organism is multi-drug-resistant in the majority of cases, and as a result, its eradication brings about a great diagnostic challenge to physicians. We hereby describe a unique and rare case of a preterm neonate admitted due to late-onset sepsis and diagnosed with a multi-drug-resistant isolate of E. meningoseptica. The objective of this case report is to identify it as a potential cause of neonatal late-onset sepsis along with its clinical, diagnostic, and therapeutic dilemmas.

## Case presentation

We present a case of a male neonate who presented to the outpatient clinic on the eleventh day of his life with a history of fever, reluctance to feed, and recurrent episodes of vomiting for three days. The baby was prematurely born at 35 weeks gestation with a low birth weight of 2,100 g from a preterm twin pregnancy to a 33-year-old primigravida mother via spontaneous vaginal delivery. The patient had the appearance, pulse, grimace, activity, and respiration (APGAR) scores of 6/10, 8/10, and 9/10 at 1, 5, and 10 minutes, respectively. To our knowledge, the other twin remained healthy throughout. On initial examination in our case, jaundice was clinically apparent. Extreme agitation and a near-inconsolable cry were noted in the neonate. Other positive findings included hyperpyrexia (102 °F), tachycardia, tachypnea, tense fontanelles, and delayed neonatal reflexes. An initial diagnosis of late-onset neonatal sepsis was made, and the patient was admitted to the neonatal intensive care unit (NICU) for careful monitoring on suspicion of concurrent meningitis. Laboratory tests were ordered, which included a complete blood picture, serum bilirubin analysis, serum urea and creatinine, serum electrolytes, and C-reactive protein. The results of these initial laboratory tests are shown in Table 1.

**Table 1:** Baseline laboratory parameters of the patient.

Parameters	Values
Hemoglobin (g/dL)	15
Total leukocyte count (μL)	5,460
Platelets (μL)	110,000
Hematocrit	44.5%
Mean corpuscular volume (fL)	98.5
Mean corpuscular hemoglobin (pg)	33.2
Polymorphs	66%
Lymphocytes	29%
Monocytes	4%
Eosinophils	1%
Bilirubin total (mg/dL)	9.9
Bilirubin direct (mg/dL)	0.4
Bilirubin indirect (mg/dL)	9.5
Urea (mg/dL)	14
Creatinine (mg/dL)	0.5
Sodium (mmol/L)	145
Potassium (mmol/L)	4.1
Chloride (mmol/L)	100
C-reactive protein (mg/L)	105.9

Simultaneously, two repeat back-to-back blood cultures (from two different sites) were drawn, which showed growth of E. meningoseptica after 24 hours of incubation. Thereafter, subsequent cultures also showed similar growth and sensitivities. The results of the sensitivity patterns are shown in Table 2.

**Table 2:** An elucidation of sensitivity patterns to various antimicrobial agents.

Antimicrobial agent	Sensitivity pattern
Tigecycline	Sensitive
Chloramphenicol	Resistant
Azithromycin	Sensitive
Vancomycin	Sensitive
Ciprofloxacin	Sensitive
Levofloxacin	Sensitive
Co-trimoxazole	Resistant
Piperacillin/Tazobactam	Resistant
Imipenem	Resistant
Meropenem	Resistant
Gentamicin	Resistant
Tetracycline	Resistant
Ceftriaxone	Resistant
Cefepime	Resistant
Colistin	Resistant
Ampicillin	Resistant
Amoxicillin/Clavulanic acid	Resistant

Within four hours of admission, the patient had an episode of focal clonic seizure involving the left upper and lower extremities that progressed into a multifocal clonic seizure involving all four extremities and the face. Nil per oral (NPO) was ordered along with maintenance intravenous fluids (0.2% saline with 10% dextrose water), and the patient was started on empirical intravenous antibiotic therapy (cefotaxime and Amikacin). Supplemental oxygen inhalation was started at a flow rate of 3 L/minute via nasal prongs due to decreasing oxygen saturation. Thereafter, a lumbar puncture was performed that yielded a yellow, turbid cerebrospinal fluid (CSF) sample. The findings of CSF analysis were strongly suggestive of bacterial meningitis. The initial findings of the CSF examination are delineated in Table 3.

**Table 3:** Initial results of the cerebrospinal fluid analysis. AFB, acid-fast bacilli

Parameters	Values
Color	Light yellow
Quantity (mL)	4
PH	7.8
Xanthochromia	Positive
Protein (mg/dL)	317
Sugar (mg/dL)	10
Leukocytes (μL)	8,000
Red blood cells	None
Polymorphs	90%
Lymphocytes	10%
Gram stain	Gram-negative rods
Ziehl-Neelsen stain	No AFB

CSF cultures and sensitivity patterns showed growth of E. meningoseptica sensitive to vancomycin. The results of CSF culture and sensitivity patterns are shown in Table 4.

**Table 4:** Sensitivity patterns obtained from cerebrospinal fluid cultures.

Antimicrobial agent	Sensitivity pattern
Tigecycline	Sensitive
Chloramphenicol	Sensitive
Azithromycin	Sensitive
Vancomycin	Sensitive
Ciprofloxacin	Sensitive
Levofloxacin	Sensitive
Co-trimoxazole	Sensitive
Piperacillin/Tazobactam	Resistant
Imipenem	Resistant
Meropenem	Resistant
Gentamicin	Resistant
Tetracycline	Resistant
Ceftriaxone	Resistant
Cefepime	Resistant
Colistin	Resistant
Ampicillin	Resistant
Amoxicillin/Clavulanic acid	Resistant

The patient's blood reports were significant for a low total leukocyte count of 5,460 μL^-1^, which depicted a poor prognosis in our case. The patient's total serum bilirubin was 9.9 mg/dL. In general appearance, the patient was clinically severely jaundiced; therefore, we started phototherapy. After 15 hours of admission, the blood culture revealed an isolate of E. meningoseptica sensitive to tigecycline, azithromycin, vancomycin, ciprofloxacin, and levofloxacin. The patient was immediately put on intravenous vancomycin (30 mg/kg/day in two divided doses) and ciprofloxacin (40 mg/kg/day in two divided doses) in meningitic doses. The use of this combination of antibiotics was reviewed and approved by an infectious disease physician. Intravenous acetaminophen was given for febrile episodes. Temporarily, the seizures were controlled using intravenous midazolam. After that, the patient was given a loading dose of intravenous phenobarbital with subsequent maintenance doses. Later, intravenous levetiracetam had to be added to the regimen to control recurrent episodes of multifocal seizures. However, electroencephalography (EEG) could not be obtained due to the nonavailability of resources. CSF analysis was positive for xanthochromia and showed elevated proteins and low glucose, raising the suspicion of bacterial meningitis. Gram staining showed numerous gram-negative rods in the CSF sample. After 28 hours of admission, CSF culture became positive for the same organism. The hospital stay was complicated by multiple episodes of hypotension and tachycardia, for which intravenous fluid boluses had to be administered.

After 48 hours of the start of appropriate antibiotics, there was an apparent clinical improvement. A nasogastric tube was inserted and a feeding trial was given through it, which the patient tolerated. Nasogastric feed was slowly and regularly built up thereafter. Intravenous anticonvulsants were changed to oral formulations given through the nasogastric tube. Phototherapy was administered intermittently throughout the treatment, which led to improved jaundice. After 72 hours, there was a complete cessation of febrile episodes. The patient tolerated an oral feeding trial. Intravenous vancomycin and ciprofloxacin were continued for one week, leading to the resolution of all symptoms. Blood samples obtained one week after the employment of appropriate antimicrobials were negative.

Due to financial constraints, the patient’s guardians opted to be discharged on request. Upon discharge, the patient was vitally and hemodynamically stable and was taking and tolerating oral feeding. There were no neurological deficits. Intravenous vancomycin and ciprofloxacin were added for an extended seven-day period as discharge medications, which were administered in outpatient antibiotic therapy every day. At the follow-up visit one month after getting discharged, the patient was stable and without any neurological deficit. The patient was followed up again two months after discharge in tele-clinic and was thriving well with no complaints. Further follow-up visits were not possible due to geographical constraints.

## Discussion

Bacteria from the genus Elizabethkingia have been isolated from water bodies, reserves, and wet soil [1]. They have also been isolated from fish [4], frogs [5], and arthropods, such as mosquitoes [6]. Elizabethkingia spp. has also been identified as an emerging cause of nosocomial infections and outbreaks, especially in the critical care setting. In accordance with that, isolates have been recovered from hospital sinks, taps, and other critical care equipment such as endotracheal tubes and bronchial aspirates of patients on mechanical ventilation [7]. The isolation of these organisms from various hospital sources may be due to their biofilm-forming ability [4].

In adults, Elizabethkingia spp. is associated with a wide array of infections such as endocarditis, cellulitis, bronchitis, epididymitis, and abdominal and eye infections [8]. E. meningoseptica has also been implicated in coinfection in COVID-19 patients [9]. Most cases reported in the adult population were hospital-acquired [8]. The most common presentations of E. meningoseptica infection in the pediatric population are either early onset sepsis or meningitis, with very few cases diagnosed as both sepsis and meningitis [10]. Regardless, E. meningoseptica is still a very rare cause of meningitis and bacteremia in the pediatric population, so much so that only 283 cases of pediatric E. meningoseptica infections were reported from 1944 to 2017 [10]. Data from this review of the limited literature available on the subject matter indicates higher mortality and morbidity in neonates as compared to infants and older children. Neonates also suffered the most in terms of neurological morbidity, as 30.4% of all survivors developed hydrocephalus and 6.5% developed some degree of hearing loss [10]. In Pakistan, only a few cases of neonatal infection/sepsis with this organism have been officially reported. The initial diagnosis of late-onset sepsis was made at the time of presentation because the symptoms started on the eighth day of life and because our patient met the clinical criteria for systemic inflammatory response syndrome (SIRS). Later on, with the addition of laboratory results and positive cultures, our patient met all the criteria for sepsis that were listed in the 2005 consensus definition for pediatric sepsis [11]. This unusual and rare presentation of E. meningoseptica with late-onset sepsis and meningitis makes our case distinct from other similarly published cases in the medical literature.

As mentioned earlier, Elizabethkingia spp. is intrinsically resistant to some antibiotics including β-lactam, β-lactam/β-lactamase inhibitor combinations, and carbapenems. This intrinsic antimicrobial resistance is due to the presence of special metallo β-lactamase (MBL) genes in the Elizabethkingia species that encode unique β-lactamases and carbapenemases [12]. The isolate obtained in our case is similar in that it was only sensitive to chloramphenicol, a tetracycline (tigecycline), macrolide (azithromycin), sulfonamide (trimethoprim/sulfamethoxazole), glycopeptide (vancomycin), and fluoroquinolone (ciprofloxacin and levofloxacin) group of antibiotics. This intrinsic antimicrobial resistance, isolation of strains with variable susceptibility patterns [2,8,10], and genomic similarity between some species of this genus [9] make these organisms difficult and tricky to diagnose [3] and treat. Moreover, the isolation of these organisms from natural resources such as water, soil, and animals [4-6] makes them a potential source of transmission of antimicrobial resistance in nature, expanding on their clinical importance.

In our case, both blood and CSF samples, drawn at admission simultaneously, showed growth of E. meningoseptica. Time to positivity for blood culture was slightly shorter (15 hours after admission) than for CSF culture (28 hours after admission). CSF positive for xanthochromia in our case may be explained by hyperbilirubinemia and/or high protein content in the CSF. There are limited studies on the role of various antibiotics against Elizabethkingia spp. According to the available data on E. meningoseptica meningitis and bacteremia, vancomycin combined with either ciprofloxacin, linezolid, or rifampicin turned out to be an effective antibacterial therapy with high cure rates [13]. Vancomycin has a high CSF penetration in the presence of meningitis [14] and has also been studied as a potential empirical antibiotic choice in patients with bacterial meningitis [15], providing fruitful results against drug-resistant and unusual organisms, especially in combination with other synergistically acting antibiotic drugs. Ciprofloxacin has also been successful in treating bacterial meningitis showing adequate CSF penetration in meningitis caused by a variety of organisms, including Streptococcus pneumoniae, Listeria monocytogenes, Enterococcus faecalis, Streptococcus milleri, Haemophilus influenzae, Pseudomonas aeruginosa, and Staphylococcus aureus. CSF concentrations of ciprofloxacin were more than or equal to the minimum inhibitory concentration of the aforementioned bacteria [16]. In our case, early positive blood culture resulted in early rigorous treatment with intravenous vancomycin and ciprofloxacin, which brought forth a good clinical outcome and prevented the development of acute neurological sequelae that are associated with E. meningoseptica meningitis and bacteremia. Any comments on delayed neurological deficits cannot be made as long-term follow-up with the patient was not possible.

## Conclusions

In recent times, the prevalence of Elizabethkingia species in causing nosocomial infections and hospital outbreaks has come to light. Despite this, available clinical and genetic data on these organisms is limited. Proper sterilization and disinfection protocols in intensive care units, both pediatric and adult, should be in place to prevent hospital-acquired infections, outbreaks, and colonization, which could lead to subsequent infection. As such, Elizabethkingia-related infections should always be considered in the differential diagnoses in cases of neonatal sepsis and/or meningitis. Vancomycin-combination regimens have shown their clinical potential in both curing and preventing sequelae associated with this organism. Hence, these regimens should be considered strongly in cases with susceptible isolates.
